# Diastolic Plateau – Invasive Hemodynamic Marker of Adverse Outcome Among Left Ventricular Assist Device Patients

**DOI:** 10.3389/fcvm.2022.847205

**Published:** 2022-04-05

**Authors:** Avishay Grupper, Afek Kodesh, Jacob Lavee, Paul Fefer, Israel M. Barbash, Dan Elian, Alexander Kogan, Avi Morgan, Amit Segev, Elad Maor

**Affiliations:** ^1^Division of Cardiology, Leviev Center of Cardiovascular Medicine, Sheba Medical Center in Tel HaShomer, Ramat Gan, Israel; ^2^The Sackler School of Medicine, Tel Aviv University, Tel Aviv, Israel

**Keywords:** hemodynamic, diastolic plateau, LVAD, right ventricular, outcome

## Abstract

**Background:**

Diastolic plateau is an invasive hemodynamic marker of impaired right ventricular (RV) diastolic filling. The purpose of the current analysis was to evaluate the prognostic importance of this sign in left ventricular assist device (LVAD) patients.

**Methods:**

The analysis included all LVAD patients who received continuous-flow LVAD (HeartMate 3) at the Sheba medical center and underwent right heart catheterization (RHC) during follow up post-LVAD surgery. Patients were dichotomized into 2 mutually exclusive groups based on a plateau duration cutoff of 55% of diastole. The primary end point of the current analysis was the composite of death, heart transplantation, or increase in diuretic dosage in a 12-month follow-up period post-RHC.

**Results:**

Study cohort included 59 LVAD patients with a mean age of 57 (IQR 54–66) of whom 48 (81%) were males. RHC was performed at 303 ± 36 days after LVAD surgery. Patients with and without diastolic plateau had similar clinical, echocardiographic, and hemodynamic parameters. Kaplan–Meier survival analysis showed that the cumulative probability of event at 1 year was 65 ± 49% vs. 21 ± 42% for primary outcomes among patients with and without diastolic plateau (p Log rank < 0.05 for both). A multivariate model with adjustment for age, INTERMACS score and ischemic cardiomyopathy consistently showed that patients with diastolic plateau were 4 times more likely to meet the study composite end point (HR = 4.35, 95% CI 1.75–10.83, *p* = 0.002).

**Conclusion:**

Diastolic plateau during RHC is a marker of adverse outcome among LVAD patients.

## Introduction

Right ventricular failure (RVF) following left ventricular assist device (LVAD) implantation remains a major complication which may significantly impair patient outcomes. It is associated with prolonged length of intensive care unit and hospital stay, as well as high long-term morbidity and mortality (1–4). Since many LVAD patients have clinical or subclinical right ventricular (RV) dysfunction, accurate RV function assessment is essential in diagnosing RVF, guiding therapies, and determining prognoses. As the RV is embryologically and morphologically distinct, non-invasive imaging tests traditionally used for the left ventricle (LV) may not be ideal in measuring RV function (5). In addition, there are no clear invasive hemodynamic criteria that can be used to define or diagnose RVF post-LVAD. The pathophysiology of RVF post-LVAD is multifactorial; however, one of the major factors affecting RV function is LVAD speed, leading to alterations of RV preload and afterload as well as positional distortions of the interventricular septum. These changes limit the flexibility and mobility of the RV myocardium and can create a restrictive-like physiology.

Dip and plateau (“square root sign”) is a classic hemodynamic parameter used to describe a typical RV pressure pattern in patients with constrictive or restrictive physiology (6, 7). An early rapid filling of the RV in early diastole due to high atrial pressure, followed by a limitation in filling from the stiff myocardium results in a prominent “y” descent on the atrial pressure curves. The pressure in late diastole elevates and plateaus in accordance with the impaired RV relaxation or pericardial compression, resulting in the “square root” sign on RV pressure curves.

While classic teaching associates the square root sign with constrictive pericarditis, cardiac tamponade, and restrictive cardiomyopathy, we hypothesized that this sign could be used to assist in identifying LVAD patients with failing RVs. Therefore, the purpose of the current study was to evaluate the role of the invasive hemodynamic dip and plateau pattern as a marker of adverse outcomes among LVAD patients.

## Materials and Methods

**FIGURE 1:**
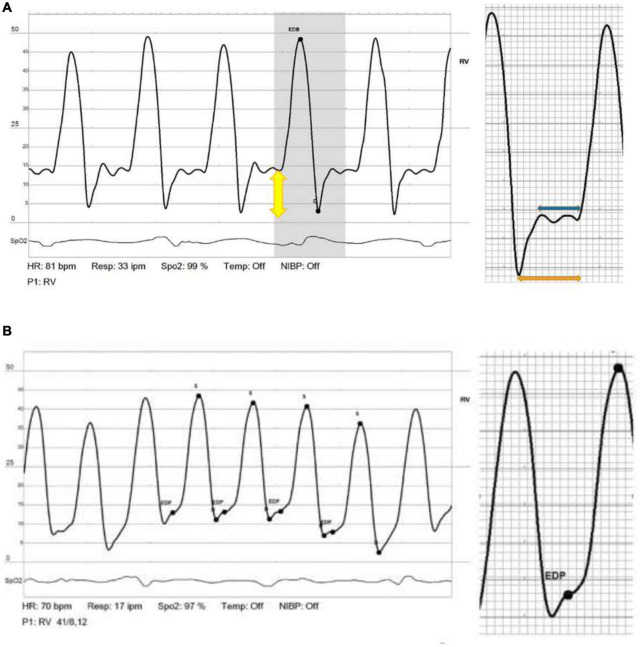
**(A)** Positive diastolic plateau. **(B)** Normal right heart tracing with no diastolic plateau.

The study included 59 consecutive patients who had undergone LVAD—HeartMate3 implantations at the Sheba medical center in Ramat Gan, Israel and underwent invasive right heart catheterization (RHC) as part of their follow up at the LVAD clinic. All patient data was taken from the computerized medical records. For patients who had more than one RHC study, the first RHC post-LVAD implantation was used to calculate the dip and plateau of the RV waveform. All measurements were based on end-expiration phase. All RV waveforms were reviewed, and the measurement was based on the best waveform (the one with the minimum number of artifacts). Plateau was calculated by dividing the length of the plateau by the length of the entire RV diastole; for dip calculations, catheterization pressure readings were used (Figure 1). Since there is no acceptable cutoff for diastolic plateau, positive plateau was defined as the plateau ≥ 55% of diastole, and the study population was dichotomized into 2 groups according to the diastolic plateau pattern (positive vs. negative plateau). This cutoff was used as it is statistically significant in multiple models (Kaplan–Meier, univariate Cox regression and multivariate Cox regression).

All reported echocardiographs were carried out based on the American Society of Echocardiography guidelines. Right atrial pressure was estimated by visualizing the inferior vena cava (IVC) and its response to respiration. Right atrial pressure was estimated as 5 mm Hg if the IVC was < 2.0 cm in diameter at the junction of the right atrium, 15 mm Hg if the IVC was dilated and collapsed with respiration, and 20 mm Hg if the IVC was dilated and did not collapse with respiration.

Outcome events used in this study included all-cause mortality, heart transplantation, and furosemide dosage increases throughout a 12-month period post index RHC (as a parameter associated with worsening RV failure signs). The primary composite outcome of the study included death, heart transplantation, or increase in diuretic dosage in a 12-month follow-up period post-RHC. Secondary composite outcome included only death and diuretic dose increase post-RHC. Mortality data was available for all patients from the national registry. Heart transplantation and furosemide dosage increases were available for all patients from the electronic medical record. The Institutional Review Board of the Sheba Medical Center approved this retrospective analysis based on strict maintenance of participants’ anonymity during database analyses. No individual consent was obtained.

### Statistical Analysis

Patient characteristics were presented as means for continuous variables and a binary system was used for categorical variables. Student *t*-test was used for comparison of continuous variables between the study groups. Pearson’s R and Spearman correlations were used for the same purpose for categorical variables. The probability of meeting the composite endpoint according to the study groups was graphically displayed according to the method of Kaplan–Meier, with a comparison of cumulative survival across strata by the log-rank test. Univariate and multivariable Cox proportional hazards regression modeling were used to determine the Hazard Ratio (HR) for the composite study endpoint. In addition to diastolic plateau groups, the multivariable model included age, INTERMACS score and ischemic cardiomyopathy. Hazards Ratio is presented with a 95% confidence interval and statistical significance was accepted for a 2-sided *P* < 0.05. All statistical analyses were performed using IBM SPSS version 23.

## Results

**FIGURE 2:**
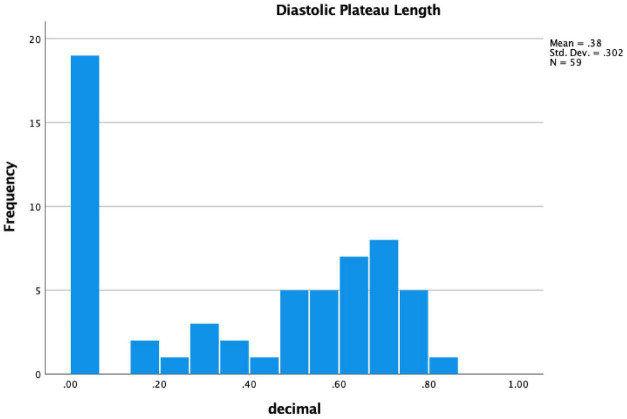
Diastolic plateau distribution.

Final study cohort included 59 LVAD patients with a mean age of 57 years (IQR 54-66), and 48 patients (81%) were male. Baseline patients’ characteristics at the time of RHC are shown in Table 1. A histogram depicting the distribution of diastolic plateau length measured at the time of RHC is shown in Figure 2. Overall, 26 (44%) patients exhibited a positive diastolic plateau and were more likely to have chronic kidney disease, defined as a GFR < 40 ml/min/1.73 m^2^, as well as lack of mineralocorticoid treatment compared to LVAD patients without diastolic plateau (*p* ≤ 0.05). Other baseline characteristics did not differ between the 2 groups (Table 1). None of the study cohort patients experienced in-hospital postoperative RVF after LVAD implantation according to the Interagency Registry for Mechanically Assisted Circulatory Support (INTERMACS) definition (8).

**TABLE 1:** Baseline patient characteristics.

	All (*N* = 59)	Diastolic Plateau (+) (*N* = 26)	Diastolic Plateau (−) (*N* = 33)	*P* value
Age, y	57	59	56	0.25
Gender (M)	48	21 (81%)	27 (82%)	0.92
INTERMACS (1)	2	1 (4%)	1 (3%)	0.75
INTERMACS (2)	14	7 (27%)	7 (21%)	0.75
INTERMACS (3)	18	7 (27%)	11 (33%)	0.75
INTERMACS (4)	25	11 (42%)	14 (42%)	0.75
Ischemic cause	31	15 (58%)	16 (48%)	0.49
Diabetes	30	15 (58%)	15 (45%)	0.36
Hypertension	18	9 (35%)	9 (27%)	0.55
Body mass index	27	27 ± 4	28 ± 4	0.26
AICD	49	21 (81%)	28 (85%)	0.69
Atrial fibrillation	26	12 (46%)	14 (42%)	0.78
History of VT	26	10 (38%)	16 (48%)	0.45
GFR, ml/min⋅1.73 m^2^	74	76	73	0.61
GFR < 40	3	3 (12%)	0 (0%)	0.05
Hgb, g/dl	12.6	12.3	12.8	0.39
Serum sodium, mEq/L	138.9	138.8	138.9	0.88
Total bilirubin, mg/dl	0.76	0.83	0.71	0.37
AST, U/L	27	25	28	0.54
ALT, U/L	24	20	26	0.1
Beta blockers	55	26 (100%)	29 (88%)	0.07
ACEI/ARBs	26	13 (50%)	13 (39%)	0.42
MRA	44	16 (62%)	28 (85%)	0.04
Furosemide	34	15 (58%)	19 (58%)	0.99

*AICD, Automatic Implantable Cardioverter Defibrillator; VT, ventricular tachycardia; GFR, glomerular filtration rate; Hgb, hemoglobin; AST, aspartate aminotransferase; ALT, Alanine transaminase; ACEI, Angiotensin converting enzyme inhibitor; ARB, angiotensin receptor blocker; MRA, Mineralocorticoid receptor antagonist.*

Echocardiography characteristics are shown in Table 2. Echo exams were performed at 7 ± 16 days from RHC without significant difference between the groups (21 ± 15 days for patients with negative plateau and 12 ± 19 days for patients with a positive plateau, *p* = 0.40). There were no statistically significant differences between patients with and without diastolic plateau in most echocardiographic parameters including RV functional assessment and pulmonary systolic pressure estimations. The inter-ventricular septum was in neutral position in all patients and no pump parameters adjustment was required during echo exams. Patients with a positive diastolic plateau were more likely to have more significant aortic regurgitation compared to patients without diastolic plateau (*p* = 0.02).

**TABLE 2:** Echocardiography characteristics.

	All (*N* = 59)	Diastolic plateau (+) (*N* = 26)	Diastolic plateau (−) (*N* = 33)	*P* value
**Baseline Pre-LVAD**				
Estimated RVSP (mmHg)	52.2	53.2	51.3	0.59
RV Size	0.44	0.40	0.47	0.61
RV dysfunction	1.19	1.11	1.24	0.63
TR degree	1.25	1.21	1.27	0.79
**Post LVAD**				
LVESD (mm)	43	43	44	0.82
LVEDD (mm)	53	53	54	0.78
Left atrium size (cm)	4.7	4.7	4.6	0.64
IVS (mm)	10.3	9.6	10.8	0.07
LV mass (gr)	205	191	217	0.27
LV mass index (gr/m^2^)	104	98	110	0.25
MR > mild	9	5 (19%)	4 (12%)	0.48
AI > mild	16	11 (42%)	5 (15%)	0.02
RV dysfunction	35	15 (58%)	20 (61%)	0.85
RV dilatation > mild	30	13 (50%)	17 (52%)	0.86
TR > mild	24	10 (38%)	14 (42%)	0.7
Estimated RA pressure (mmHg)	12	11	12	0.45
Estimated RVSP (mmHg)	31	30	33	0.19
Pump Speed (RPM)	5602	5635	5576	0.40

*RVSP, right ventricular systolic pressure; RV, right ventricle; TR, tricuspid regurgitation; LVESD, left ventricular end systolic diameter; LVEDD, left ventricular end diastolic diameter; IVS, inter-ventricular septum; LV, left ventricle; MR, mitral regurgitation; AI, aortic insufficiency; RA, right atrium. RV Size: 0 = normal, 1 = dilated; RV Function: 0 = normal, 1 = mildly reduced, 2 = moderately reduced, 3 = severely reduced; TV Function: 0 = trivial; 1 = mild; 2 = moderate; 3 = severe.*

RHC was performed at 303 ± 36 days after LVAD implantation surgery. There was no significant difference at the mean time from LVAD surgery to RHC between the groups (291 ± 33 days for patients with negative plateau vs. 318 ± 38 days for patients with positive plateau, *p* = 0.647). RHC data are shown in Table 3 with no statistically significant differences in all hemodynamic parameters between study groups. Hence, there was no need for LVAD parameters adjustment during RHC.

**TABLE 3:** Right heart catheterization data.

	All (*N* = 59)	Diastolic Plateau (+) (*N* = 26)	Diastolic Plateau (−) (*N* = 33)	*P* value
RA mean (mmHg)	14	14	13	0.9
RVEDP (mmHg)	7.4	7	7.7	0.66
PA mean (mmHg)	23	23	24	0.55
PA systolic (mmHg)	35	35	35	0.98
PA diastolic (mmHg)	17	16	18	0.1
PCWP (mmHg)	14	13	15	0.18
TPG	10	10	9	0.13
Diastolic TPG	3.3	3.3	3.2	0.93
PVR (WU)	2.3	2.2	2.4	0.68
Cardiac output, l/min	4.2	4.2	4.3	0.74
Cardiac index, L/min/m^2^	2.2	2.2	2.1	0.7
Pump Speed (RPM)	5602	5635	5576	0.40
Heart rate (BPM)	78	79	76	0.32

*RA, right atrium; RVEDP, right ventricular end diastolic pressure; PA, pulmonary artery; PCWP, pulmonary capillary wedge pressure; TPG, trans-pulmonary gradient; PVR, pulmonary vascular resistance.*

**FIGURE 3:**
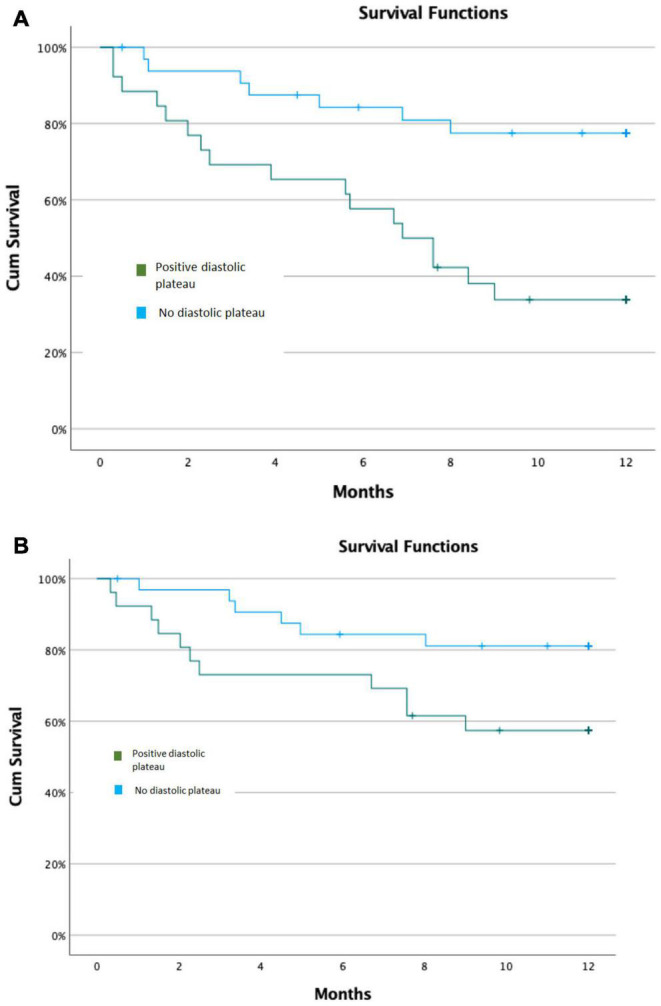
Kaplan–Meier Survival Models. **(A)** Kaplan–Meier Survival Model for primary composite outcomes. **(B)** Kaplan–Meier Survival Model for secondary composite outcomes.

Kaplan–Meier survival analysis demonstrated that the cumulative probability of meeting the primary composite endpoint at 12 months was 65 49% among LVAD patients with a positive diastolic plateau vs. 21 42% among those without a diastolic plateau (p log rank < 0.001) (Figure 3A). The cumulative probability of meeting the secondary composite endpoint at 12 months for patients with positive plateau was 42 50% compared to 18 39% for those with negative plateau (p log rank = 0.043) (Figure 3B).

The distribution of outcome events during follow up was as follow: 7/33 patients without diastolic plateau presented with positive end points: (4 diuretic dose increase, 2 heart transplant, 1 death); 17/26 patients with diastolic plateau presented with positive end points: (7 diuretic dose increase, 7 heart transplant, 3 death). Overall, 11 outcome events due to increase diuretic dose vs. 13 outcome events due to heart transplant or mortality.

Cox regression survival analysis with adjustment for age, INTERMACS score and ischemic cardiomyopathy showed an independent association of the diastolic plateau with primary study outcomes such that patients with positive diastolic plateau were 4 times more likely to reach the study end points (HR = 4.35, 95% CI 1.75–10.83, *p* = 0.002). The significant association was consistent for secondary composite outcomes as well, such that patient with positive diastolic plateau were 3 times more likely to suffer study endpoint (HR = 2.96, 95% CI 1.04–8.41, *p* = 0.041).

In contrast to the diastolic plateau, hemodynamic “dip” pattern was not significantly associated with event outcomes in the study population. On average, patients that did not suffer an event had dips of 7.5 mmHg according to their RHC while patients who did suffer an event had an average dip of 8.1 mmHg (*p* = 0.37).

## Discussion

The main finding of the current analysis is that diastolic plateau, a classic hemodynamic sign of impaired right heart filling, is associated with adverse outcomes among LVAD patients. Our data demonstrated a significant association between positive diastolic plateau, measured during an ambulatory RHC of LVAD patients, and increased risk for the combination of mortality, heart transplantation, and the need for diuretic therapy augmentation. Furthermore, adjusted survival analysis showed that LVAD patients with positive diastolic plateau were 4 times more likely to reach the study end points after adjustment for age, INTERMACS score and ischemic HF etiology.

Despite technological applications and evolving surgical experience with LVAD, the incidence of RHF is ranging from 10 to 40% after LVAD therapy, and approximately 6–10% of patients require right ventricular assist device support (1–4, 9, 10). Post-LVAD RHF remains a significant reason for morbidity and mortality and is associated with more than a 20% reduction in perioperative survival (1–4). The mechanisms underlying RVF post-LVAD are often multifactorial. Alterations in RV geometry with septal distortion due to high LVAD speed, exacerbations of pre-existing RV failure due to sudden increases in venous return and RV preload induced by the improvement in cardiac output, additional intra-operative RV injury, and increased RV sensitivity to afterload over time can all lead to RVF (11, 12). However, while RVF was initially felt to be an early post-operative complication, development of late RVF has been more frequently described (13). Late-onset RVF can manifest several months to years after device implantation and has significant adverse prognostic implications for patient outcomes (13). Hence, diagnosing RVF post-LVAD is paramount in patient management, which is established by adjusting LVAD speed, tailoring diuretic therapy, and determining a patient’s status for heart transplant candidacy.

Although Transthoracic echocardiography (TTE) is routinely utilized for assessment of RV size and function, post-LVAD evaluation of RV dimensions and function by TTE may be technically difficult. This is not only because of the intrinsic complex RV geometry, but also because post-operative changes and device-related artifacts limit visualization and accuracy of ultrasound-based measurements (5). RHC, however, remains an important tool in post-LVAD follow up, providing direct hemodynamic measurements that can be used to determine cardiac chambers filling pressures, cardiac output, and vascular resistance. Hemodynamic testing has been shown to be effective in guiding patient management and reducing adverse events, even in apparently stable and well-compensated LVAD patients (14, 15).

To date, the role of hemodynamic dip and plateau measurements have not been investigated in LVAD patients. Various studies have implicated changes in the RV during LVAD implantation process with prolonged mechanical circulatory support. These include damage to the RV during surgery, disadvantageous changes in ventricular interdependence mediated by reduced LV contractility, changes in septal architecture, and alterations in RV shape, which may create a restrictive-like RV physiology (11, 16). The primary hemodynamic consequence of restriction is the limitation of the total volume of blood that can be accommodated by the heart during diastole. Accentuated early rapid ventricular filling occurs due to increase preload and improved cardiac output with LVAD support, followed by a sudden rapid rise in pressure from the RV. These filling pressures are confined by the interventricular septum and by the geometric changes post-LVAD that limit myocardial stretching during diastole. These changes may account for the “square root” sign on ventricular pressures.

Imamura et al. reported an association between deep y-descent on RHC waveform at 6 months post-LVAD implantation and LVAD related complications (gastrointestinal bleeding, stroke, or pump thrombosis) (17). Our study, however, evaluated both components of the diastolic pressure curves (dip and plateau) and demonstrated a significant association between positive diastolic plateau and, more specifically, RVF related outcomes (mortality and diuretic therapy augmentation). Although our findings did not demonstrate a significant association between hemodynamic dip and event outcomes, all our study population had a y descent deeper than 3 mmHg (which was the value used for analysis in the study by Imamura et al.). In addition, the prominent y descent on the pressure curves represents early rapid filling of the ventricles in early diastole due to high atrial pressures of increased preload, while positive diastolic plateau pattern is often seen in ventricular restrictive physiology (6, 7). Our results showing similar right atrial pressure in both groups may explain the lack of association between hemodynamic dip and patients’ outcome and highlight the role of diastolic plateau as a more specific marker for RV diastolic dysfunction.

Importantly, there were no significant differences in most clinical, laboratory, echocardiographic, or hemodynamic parameters among patients with or without diastolic plateau in our study population, which may suggest this hemodynamic parameter is an earlier sign for RVF and clinical deterioration among LVAD patients. The decreased kidney function among LVAD patients with positive diastolic plateau may suggest a cardio-renal effect as another early sign of RVF post-LVAD (18). Furthermore, the present study suggests that even a hemodynamic snapshot can be used as a clinical marker to identify possible RV dysfunction. This can serve to guide LVAD patient management and can be an improvement to the standard TTE examination.

### Study Limitations

This analysis has all the inherent limitations of a small-size, single-center, retrospective study. Due to the single-center nature of this study and the small number of patients included, generalization of the results should be applied with caution before confirmation is available from larger population analyses. Our cohort has a potential patient selection bias, as our study included LVAD patients who were able to perform RHC during follow up post LVAD surgery. All RHC studies did not include volume challenge or any other provocation test. Our study design included a relative short follow up of 12 months post RHC and although the data were collected prospectively, our study is limited by its retrospective design.

### Conclusion and Clinical Implications

The current study is the first to report the association between invasively measured diastolic plateau and adverse outcomes among LVAD patients. Our findings identify diastolic plateau as a parameter associate with increased risk for future RV failure before diagnosed by echocardiographic or hemodynamic studies. Due to the challenges in evaluating RV function in LVAD patients, our findings encourage clinicians to carefully evaluate diastolic plateau during RHC in LVAD patients in the real-life scenario, and once identify, to consider closer surveillance with more frequent studies for early diagnosis of clinical RV failure, and to adjust medical therapy as needed. Larger studies are warranted to validate our findings.

## Data Availability Statement

The raw data supporting the conclusions of this article will be made available by the authors, without undue reservation.

## Ethics Statement

The studies involving human participants were reviewed and approved by the Institutional Review Board of the Sheba Medical Center. Written informed consent for participation was not required for this study in accordance with the national legislation and the institutional requirements.

## Author Contributions

AG and EM contributed to conception and design of the study, analysis and interpretation of the data, drafting of the manuscript, revising it critically before submission, and responsible for the overall content as guarantor. AfK contributed to analysis and interpretation of data, critically revising the manuscript, and final approval of the manuscript submitted. JL, PF, IB, DE, AlK, AM, and AS critically revised the manuscript and approved the final version of the manuscript submitted. All authors contributed to the article and approved the submitted version.

## Conflict of Interest

The authors declare that the research was conducted in the absence of any commercial or financial relationships that could be construed as a potential conflict of interest.

## Publisher’s Note

All claims expressed in this article are solely those of the authors and do not necessarily represent those of their affiliated organizations, or those of the publisher, the editors and the reviewers. Any product that may be evaluated in this article, or claim that may be made by its manufacturer, is not guaranteed or endorsed by the publisher.
